# *Ikaros* expression is associated with an increased risk of chronic graft-versus-host disease

**DOI:** 10.1038/s41598-023-35609-3

**Published:** 2023-05-25

**Authors:** A. D. Pereira, V. C. de Molla, A. R. B. M. Fonseca, L. Tucunduva, Y. Novis, M. S. Pires, A. F. Popi, C. A. Arrais-Rodrigues

**Affiliations:** 1 Universidade Federal de São Paulo, São Paulo, SP Brazil; 2 Centro de Oncologia, Hospital Sírio Libanês, Rua Dona Adma Jafet, 91, São Paulo, SP 01308-050 Brazil

**Keywords:** Haematopoietic stem cells, Prognostic markers

## Abstract

Immune reconstitution after hematopoietic stem cell transplantation (HSCT) is a complex and extremely variable process. The *Ikaros* transcription factor plays an important role in hematopoiesis in several cell lines, especially in the lymphoid lineage. We hypothesized that *Ikaros* might influence immune reconstitution, and consequently, the risk of opportunistic infections, relapse, and graft versus host disease (GVHD). Samples were collected from the graft and from the peripheral blood (PB) of the recipients 3 weeks after neutrophil recovery. Real-time polymerase chain reaction (RT-PCR) was performed to analyze the absolute and relative *Ikaros* expression. Patients were divided into two groups, according to *Ikaros* expression in the graft and in the recipients’ PB based on the ROC curves for moderate/severe cGVHD. A cutoff of 1.48 was used for *Ikaros* expression in the graft, and a cutoff of 0.79 was used for *Ikaros* expression in the recipients’ PB. Sixty-six patients were included in this study. Median age of patients was 52 years (range 16–80 years), 55% of them were male, and 58% of them had acute leukemia. Median follow-up period was 18 months (range 10–43 months). There was no association between *Ikaros* expression and the risk of acute GVHD, relapse, or mortality. However, a significant association was observed with the risk of chronic GVHD. Higher *Ikaros* expression in the graft was associated with a significantly higher cumulative incidence (CI) of moderate/severe chronic GVHD according to the National Institute of Health (NIH) classification at two years (54% vs. 15% for patients with lower expression, P = 0.03). A higher *Ikaros* expression in the recipients’ PB 3 weeks after engraftment was also associated with a significantly higher risk of moderate/severe chronic GVHD (65% vs. 11%, respectively, P = 0.005). In conclusion, *Ikaros* expression in the graft and in the recipients’ PB after transplantation was associated with a higher risk of moderate/severe chronic GVHD. *Ikaros* expression should be evaluated in larger prospective trials as a potential biomarker for chronic GVHD.

## Introduction

Allogeneic hematopoietic stem cell transplantation (HSCT) is a potentially curative therapy approach for several malignant and non-malignant diseases and, in some cases, the only with curative intent^1^. Unfortunately, non-relapse mortality is still very high, mainly due to infections and acute and chronic graft-versus-host disease (aGVHD and cGVHD, respectively)^2,3^. cGVHD can affect up to 50% of patients and is also responsible for significant comorbidities and low quality of life after HSCT^4^. The diagnosis of chronic GVHD is based on specific clinical features, although not all patients exhibit these signs and symptoms, and other nonspecific features may be the main manifestation. In doubtful cases, there are only a few laboratory tests that may be useful for diagnosis^5^. Several biomarkers have been studied to help establish and predict diagnosis and prognosis of cGVHD; however, to date, no biomarkers have been validated for clinical practice^6,7^.

*Ikaros* transcription factor could be a good candidate as a prognostic biomarker for the risk of cGVHD. *Ikaros* is a member of a family of zinc finger transcription factors encoded by IKZF1 gene. It is an essential regulator of hematopoiesis^8^, with an important role in T and B cell differentiation and their mature cell function^9–11^, as well as in cells of the myeloid lineage, in erythroid and neutrophil differentiation^12,13^.

IKZF1 haploinsufficiency due to germline mutations can be responsible for common variable immunodeficiency with a decrease in B cell lymphocytes, but can also lead to a more pronounced immunodeficiency with low eosinophils, neutrophils, and myeloid dendritic cells, and a dysfunction in T cells and monocytes^14,15^. As an essential hematopoietic transcription factor implicated in lymphocyte and myeloid differentiation, IKZF1 activity may be a critical component in immune reconstitution and in acute and cGVHD pathophysiology. To our knowledge, IKZF1 expression has not yet been studied in the context of HSCT. In the present study, we explored whether IKZF1 expression in mononuclear cells in the graft and in the recipients’ peripheral blood (PB) after engraftment could be associated with the risk of aGVHD or cGVHD.

## Patients and methods

### Study population

This was a non-interventional prospective study that included patients older than 16 years who underwent allogeneic HSCT between January 2017 and January 2020 in two transplant centers, Hospital Sirio-Libanes and Hospital Sao Paulo, both in Sao Paulo, Brazil. Conditioning regimen, graft source, GVHD prophylaxis, time to transplantation, and all other clinical decisions were made according to each center’s guidelines. Prophylaxis, diagnosis and treatment of GVHD are based on established consensus and do not differ between the two centers.

All subjects provided written informed consent prior to enrollment. The study was conducted in accordance with the Declaration of Helsinki and was approved by the research ethics committees of each center (CEP-UNIFESP of Hospital Sao Paulo and CEPesq/HSL of Hospital Sirio-Libanes).

### Sample preparation and analysis

All blood samples were prospectively collected. Samples were taken from the graft immediately before infusion on the day of the transplant (graft) and from the recipients’ PB 3 weeks after engraftment (engraftment + 21). Initially, our goal was to identify a readily detectable and cost-effective biomarker that could have practical applications in our country of origin where cost is a common practical limitation. In prior studies conducted by our team, we found that a three-week post-transplantation period was adequate to identify immune reconstitution, which strongly correlated with transplant outcomes^16,17^. We observed stronger correlations between the analyzed biomarkers and the outcomes from samples obtained 3 weeks after transplant compared to samples obtained at other post-transplant time-points.

Mononuclear cells were separated and stored according to institutional guidelines. Briefly, the collected material was immediately sent for freezing at the laboratory of the Institute of Education and Research at Hospital Sírio-Libanês. All collected material was individually processed to extract peripheral blood mononuclear cells (PBMCs) from each sample. Using Ficoll-Paque (SigmaAldrich, Darmstadt, Germany) as the separation gradient, the samples were centrifuged to form a buffy coat layer above the gradient. The cells in the buffy coat were separated at room temperature using a sterile pipette and subjected to three cycles of washing and resuspension in sterile phosphate buffered saline (PBS), with centrifugation between washes. The material from each patient was then preserved in a fetal bovine serum solution with 10% dimethyl sulfoxide (DMSO), stored in vials, and gradually frozen in a glycerol box at − 20 °C and subsequently at − 80 °C. Finally, they were stored in a liquid nitrogen tank until used in all subsequent tests. *Ikaros* expression was measured using real-time PCR. Total RNA was extracted from mononuclear cells using PureLink™ Micro (Thermo Fisher Scientific, Waltham, MA) or llustra RNAspin Mini (GE Healthcare Life Sciences, Chicago, IL) reagent and cDNA transcripts were quantified using the Superscript III Cells Direct cDNA (Life Technologies, Carlsbad, CA) kit. Reactions were amplified using a 7500 Fast Real-Time PCR System (Life Technologies, Carlsbad, CA) using TaqMan probes (Thermo Fisher Scientific, Waltham, MA), according to the manufacturer’s instructions. The value of 2^−ΔΔCt^ was used to calculate the fold change in gene expression, according to the studies of Schmittgen et al. and Vandesompele et al.^18,19^.

### Endpoint definitions and statistical analysis

The primary endpoint was the correlation between *Ikaros* expression and the incidence and severity of cGVHD. Secondary endpoints include the correlation between *Ikaros* expression and the incidence and severity of aGVHD, as well as overall survival (OS), progression-free survival (PFS), relapse incidence (RI), and non-relapse mortality (NRM). The severity of aGVHD was graded based on the Mount Sinai Acute GVHD International Consortium^20^, while cGVHD was scored as mild, moderate, or severe according to NIH standards^5^. Patients’ comorbidities and disease risk were classified as previously published^21,22^.

The median *Ikaros* relative expression and receiver operating characteristic (ROC) curves were used to divide patients into two groups based on high or low *Ikaros* expression levels in both the graft and the PB after engraftment, and to correlate these results with the presence of aGVHD and cGVHD.

PFS and OS probabilities were calculated using the Kaplan–Meier method and compared using the log-rank test. Cumulative incidence (CI) rates were calculated for aGVHD, cGVHD, NRM, and relapse/progression, with death considered a competing event. Ninety-five percent confidence intervals (95% CIs) were estimated using the Greenwood formula. Adjusted probabilities for outcomes after transplantation were estimated using the Cox proportional hazards method (PFS and OS) and the Fine-Gray risk regression model (aGVHD, cGVHD, NRM, and relapse/progression). The statistical analyses were performed using SPSS version 20 (SPSS Inc., Chicago, IL), R version 4.2.3 (R Foundation for Statistical Computing, Vienna, Austria, 2023; https://www.R-project.org/), and RStudio version 2023.03.0 + 386 'Cherry Blossom' (RStudio, PBC, Boston, MA; http://www.rstudio.com/).

## Results

### Patients and graft demographics

Between January 2017 and January 2020, 95 patients underwent allogeneic HSCT at the two transplant centers. Chimerism data were evaluated during first 3 months after HSCT. Full donor chimerism was defined as the presence of more than 95% of cells of donor origin. Patients who did not achieve neutrophil recovery or had neutrophil recovery but not full donor chimerism (n = 17), those who died 1 week after engraftment or earlier (n = 10), or those who were lost to follow-up (n = 2) were excluded from the analyses. There were no significant differences between the included and excluded patient groups regarding any of the clinical features (data not shown).

A total of 66 patients were included in final analysis, 43 from Hospital Sirio-Libanes and 23 from Hospital Sao Paulo. The main patient characteristics are presented in Table 1. Among patients included, 55% were male, and the median age at the time of transplant was 52 years (range 16–80 years). The HCT comorbidity index was 0 or 1 in all but four patients. The diagnosis was acute myelogenous leukemia (AML) in 41%, ALL in 17%, MDS/MPN in 20%, lymphoma in 12%, and aplastic anemia in 10% of patients. The disease risk index was low/intermediate in 83% of the patients. Most of the patients (77%) received grafts from mobilized PB stem cells and underwent reduced-intensity conditioning regimens (82%). Donors were haploidentical in 48% of cases, matched related in 29%, and matched unrelated in 23%. GVHD prophylaxis consisted of a regimen containing cyclosporine, mycophenolate mofetil, and post-transplant cyclophosphamide in 58% of cases, and anti-thymocyte globulin was used in 26% of cases. The median follow-up period was 18 months (range 10–43 months).

**Table 1:** Overview of patient characteristics.

*N*	66
Age, years (range)	52 (16–80)
Male sex, n (%)	36 (55)
Diagnostic, n (%)	
AML	27 (41)
ALL	11 (17)
MDS/MPN	13 (20)
Lymphoma	8 (12)
Aplastic anemia	7 (10)
Graft source, n (%)	
Bone marrow	15 (23)
Mobilized peripheral blood	51 (77)
Donor, n (%)	
Haploidentical	32 (48)
Related	19 (29)
Unrelated	15 (23)
Reduced conditioning intensity, n (%)	54 (82)
Use of total body irradiation, n (%)	38 (58)
Use of anti-thymocyte globulin, n (%)	17 (26)
Graft versus host disease prophylaxis, n (%)	
CsA + MMF + Post-Cy	38 (58)
CsA + MMF	22 (33)
CsA + MTX	6 (9)
Median follow up, months (range)	18 (10–43)

*ALL* acute lymphoblastic leukemia, *AML* acute myeloid leukemia, *CsA* cyclosporine, *GVHD* graft-versus-host disease, *MDS* myelodysplastic syndrome, *MMF* mycophenolate mofetil, *MPN* myeloproliferative neoplasms, *MTX* methotrexate.

Median *Ikaros* relative expression in mononuclear cells in the graft sample was 0.298 (range 0.002–25.683), while median *Ikaros* relative expression in mononuclear cells from the recipients’ engraftment + 21 samples was 0.073 (range 0.002–1.870).

There was no significant difference in the median of *Ikaros* relative expression between grafts obtained from bone marrow (0.536) or mobilized PB stem cells (0.298, P = 0.66).

The relative expression of *Ikaros* in the engraftment + 21 samples was not significantly different between patients who received post-transplant cyclophosphamide (median, 0.081) and those who did not (median: 0.072; p = 0.61). There was also no significant difference in the relative expression of *Ikaros* in the engraftment + 21 samples of patients who received antithymocyte globulin compared to those who did not (median, 0.085 vs. 0.021, respectively, P = 0.18).

Patients were then divided into two groups, according to *Ikaros* expression in the graft and in the recipients’ PB based on the ROC curves for moderate/severe cGVHD. A cutoff of 1.48 was used for *Ikaros* expression in the graft, and a cutoff of 0.79 was used for *Ikaros* expression in the recipients’ PB.

**Figure 1:**
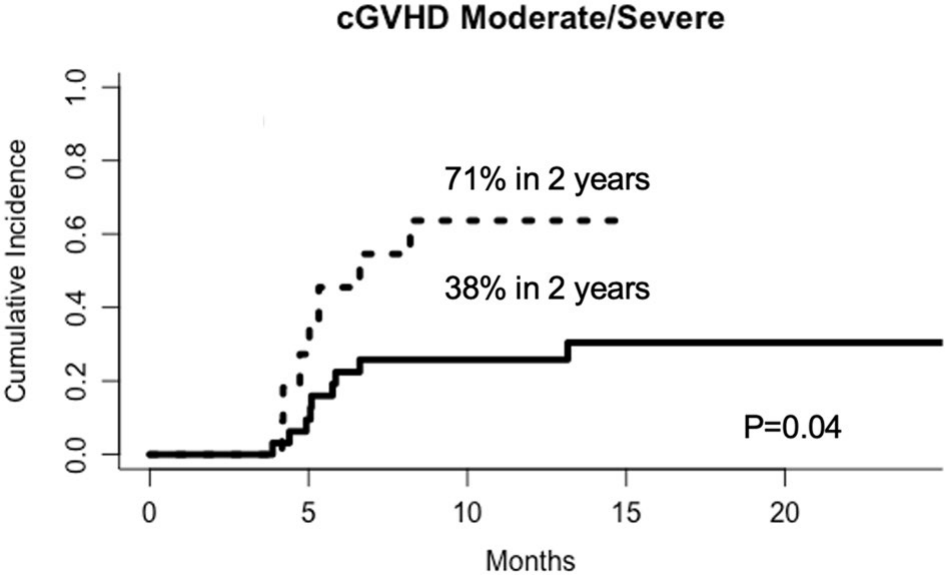
Cumulative incidence of moderate/severe cGVHD in patients with higher (dotted line) or lower (solid line) *Ikaros* expression in the graft. In the multivariate analysis, after correction for source of hematopoietic progenitor cells, use of thymoglobulin, and post-transplant cyclophosphamide, the expression of *Ikaros* did not remain an independent risk factor.

**Figure 2:**
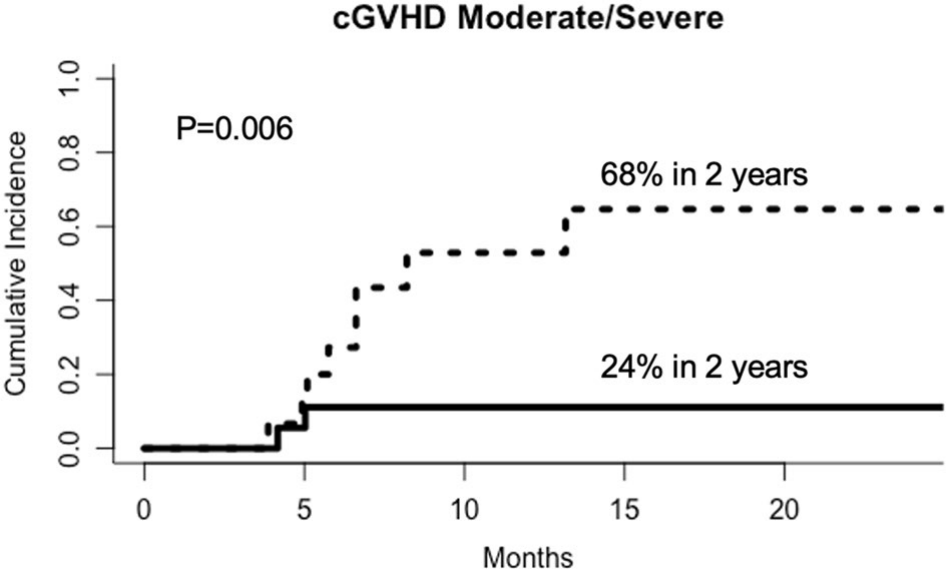
Cumulative incidence of moderate/severe cGVHD in patients with higher (dotted line) or lower (solid line) *Ikaros* expression in the patients’ peripheral blood samples 21 days after engraftment.

There was no difference in aGVHD and overall cGVHD between groups. However, the CI of moderate/severe cGVHD was significantly higher in patients with higher *Ikaros* expression in the graft (71%) than in patients with lower expression in the graft (38%, P = 0.04, Fig. 1). In addition, the CI of moderate/severe cGVHD was also significantly higher in patients with higher *Ikaros* expression in the recipients’ engraftment + 21 samples (68%) than in patients with lower *Ikaros* expression (24%, P = 0.006, Fig. 2).

**Figure 3:**
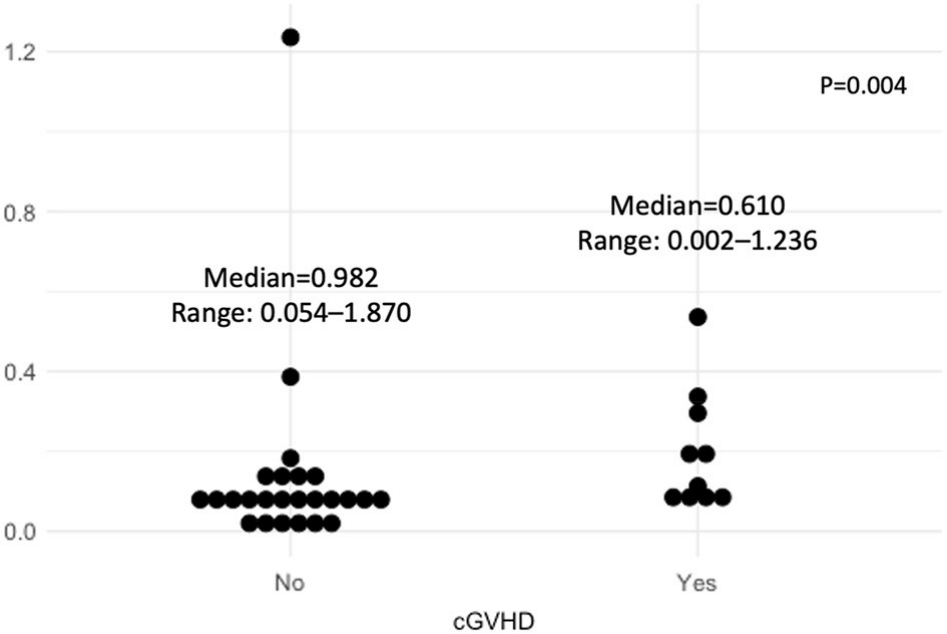
Relative expression of *Ikaros* in the patients’ peripheral blood samples 21 days after engraftment in patients with (yes) or without (no) moderate/severe cGVHD diagnosis.

The median *Ikaros* relative expression in the engraftment + 21 samples was significantly higher in patients with moderate/severe cGVHD (0.982; range 0.054–1.870) compared to patients without this complication (0.610; range 0.002–1.236, P = 0.004, Fig. 3).

In multivariate analysis, higher *Ikaros* expression in the graft did not remain statistically associated with an increased risk of moderate/severe cGVHD after adjusting for stem cell source (PB vs. bone marrow), use of ATG or post-transplant cyclophosphamide (hazard ratio = 1.82; 95% confidence interval 0.79–4.19; P = 0.16).

However, higher *Ikaros* expression in the engraftment + 21 sample remained an independent risk factor for moderate/severe cGVHD (hazard ratio = 2.84; 95% confidence interval 1.10–7.34; P = 0.03), after adjusting for the same covariates.

There was also no significant association between *Ikaros* expression in the graft or in the recipients’ engraftment + 21 samples for the other analyzed outcomes (OS, PFS, relapse/progression, and NRM).

## Discussion

In the present study, we identified that a higher *Ikaros* expression in mononuclear cells in the recipients’ PB after engraftment is correlated with a higher risk of moderate/severe cGVHD.

Despite all advances in HSCT in recent years, including significant improvements in survival, cGVHD remains the most important cause of long-term morbidity and mortality^23^. The management of post-transplant immunosuppression is extremely difficult due to the delicate balance between the risks of opportunistic infections and relapse, and the risk of GVHD^24^. Although several studies have investigated possible biomarkers for cGVHD, none are yet available for daily clinical practice^25^. Moderate/severe cGVHD, which was associated with a higher *Ikaros* expression in our study, is generally treated through systemic immunosuppression and carries a high risk of morbidity and mortality. Predicting the risk of moderate/severe cGVHD is of particular interest as a possible biomarker because the possibility of early intervention might be effective in reducing associated long-term morbidity and complications. On the other hand, biomarkers are probably less important for mild cGVHD, since they are not associated with an increased risk of serious complications and are generally associated with the beneficial graft-versus-tumor effect^26^.

In our study, we did not observe any association between *Ikaros* expression and the risk of aGVHD, whose pathophysiology differs significantly from that of cGVHD^27,28^. In aGVHD, there is an initial phase with tissue damage due to the conditioning regimen used and/or the presence of infectious complications, which causes the activation and proliferation of donor T lymphocytes stimulated by antigen-presenting cells, followed by an effector phase dependent on cellular and soluble inflammatory mediators such as TNF-α, IFN-γ, and IL-1 which causes tissue damage and activation of downstream pro-inflammatory pathways^27,28^. In contrast, cGVHD pathophysiology is characterized by a deficiency in the immune tolerance system, such as T and B cells, involved in chronic inflammatory activity, with subsequent development of fibrosis and, therefore, a wide variety of organs and tissues may be affected^24^. Some risk factors for cGVHD, such as the source of hematopoietic stem cells^29^ and the use of anti-thymocyte globulin, are less important for aGVHD^30,31^. Retrospective studies have shown that haploidentical stem cell transplantation with high-dose cyclophosphamide post-transplant also had lower rates of cGVHD, with no difference in the risk of aGVHD when compared to related and unrelated donors^32,33^. These data support the idea that graft characteristics and events in the early phase after graft infusion might play an important role in cGVHD, and *Ikaros* expression might be a significant contributing factor to this pathophysiology.

To the best of our knowledge, this is the first study that has explored the role of *Ikaros* in immune reconstitution after HSCT. *Ikaros* is one of the most important transcription factors involved in hematopoiesis regulation^8^ and it influences differentiation of several cell lines, including lymphoid and myeloid cell lines^11,13–15,34^, with particular importance in B cell lymphoid development^8,9^.

B cell lymphocytes and the presence of alloreactive antibodies can be an essential part of cGVHD pathophysiology^35,36^. Female donor-to-male receptor is a well-established risk factor for cGVHD, in part due to antibodies directed against epitopes encoded on the Y chromosome^37^. In addition, B cell activation factor (BAFF) is generally elevated in cGVHD, resulting in the rescue of autoreactive B lymphocytes, providing them with greater activity^38–40^. *Ikaros* can contribute to this increase in BAFF-induced B-cell activation. Patients with systemic lupus erythematosus also have elevated levels of BAFF, which leads to greater activation and proliferation of B lymphocytes. An in vitro study showed that the reduction of *Aiolos* and *Ikaros* reduced this effect of BAFF^41^.

In our study, we could not identify the specific cell type responsible for this increased *Ikaros* expression. Future studies focusing on specific analyses of B cells, CD4 and CD8 T cell lymphocytes, monocytes, dendritic cells, or other cells, could address this topic. On the other hand, the simpler collection and analysis procedures presented in this study could be more easily replicated and even used in clinical practice.

Multivariate analysis confirmed the statistical significance of the engraftment + 21 but not the graft sample. With a small number of patients, it could be hypothesized that the sample size was not sufficient to identify the statistical significance of the graft. It can also be assumed that the graft, which has not yet been exposed to host antigens, may not have sufficient stimulus for the activation of Ikaros. Further studies should be conducted to confirm the relevance of Ikaros expression in the graft.

Among the previously described biomarkers for cGVHD, only a few were collected from the graft and in the early phase after transplantation, as in our study. This characteristic is particularly favorable for decision-making during the management of immunosuppression after HSCT. One of the main risk factors for moderate/severe cGVHD is progression after aGVHD^42^. In patients with elevated *Ikaros* expression, aGVHD treatment could be intensified, given that they already have a higher risk of cGVHD. Another possible intervention could be the intensification of prophylactic immunosuppression, such as the addition of sirolimus to standard cyclosporine plus mycophenolate mofetil-based prophylaxis, which has been shown to result in lower cGVHD rates without higher relapse rates^43^. Possibly, with a better stratification of patients’ GVHD risk, we can move from the actual “one size fits all” kind of GVHD prophylaxis to a more personalized strategy. Donor selection may also be improved if the patient has more than one donor available, assuming that the donor's baseline *Ikaros* expression also influences cGVHD, a strategy that could be investigated in a prospective trial in the future.

Our study has several limitations. Initially, the small number of cases reduces the power of subgroup analysis, as discussed above. Another concern was pre-analytical laboratory errors, as samples were frozen for later analysis. To minimize the possible risk of interfering with *Ikaros* expression, samples were frozen for the shortest possible time after collection. In addition, the same technician processed the samples both during freezing and in the final analysis. The higher *Ikaros* expression in the graft than in the PB after engraftment provides evidence that the laboratory analysis is representative of its activity in vivo. The graft is an environment with greater cell proliferation and differentiation and it is expected to have significantly higher *Ikaros* expression than in the PB after engraftment. Additionally,, while our study cohort is very heterogeneous, it is quite comparable to other case series in the literature, including those analyzing data on cGVHD^44^. We had a slightly higher number of haploidentical transplants than most of the previous studies, but this seems to be a trend worldwide^44^. The limited number of each type of transplant including related, unrelated, and haploidentical, prevented a comprehensive subgroup analysis, which should be addressed in future studies. There is also an excessive number of reduced-intensity conditioning regimens, largely explained by an institutional protocol that prospectively analyzed the results of exclusively reduced-intensity conditioning regimens in one of the transplant centers. Additionally, all patients diagnosed with lymphoproliferative diseases, SMD/MPN, or aplastic anemia only received RIC regimens at their physician’s discretion. As we had a low number of patients who received myeloablative conditioning regimens, the possible effects of *Ikaros* expression in this scenario remain unclear.

In conclusion, a higher *Ikaros* expression in mononuclear cells in the PB after engraftment was significantly correlated with a higher risk of moderate/severe cGVHD, supporting its use as a prognostic biomarker. Further studies should be conducted to confirm these findings and to identify how to incorporate this marker in clinical practice.

## Data Availability

The dataset used and analyzed during the current study is available from the corresponding author on reasonable request.
